# Investigating health effects in a community surrounding a road tunnel stack – a cross sectional study

**DOI:** 10.1186/1476-069X-7-46

**Published:** 2008-09-26

**Authors:** Adam Capon, Vicky Sheppeard, Katie Irvine, Bin Jalaludin, Michael Staff, Guy Marks, Alan Willmore

**Affiliations:** 1 Environmental Health Branch, New South Wales Health Department, Australia; 2 NSW Biostatistical Officer Training Program, New South Wales Health Department, Australia; 3 Centre for Research, Evidence Management and Surveillance, Sydney South West Area Health Service, Australia and School of Public Health and Community Medicine, University of New South Wales, Australia; 4 Woolcock Institute Medical Research, Sydney, Australia; 5 Centre for Epidemiology and Research, New South Wales Health Department, Australia

## Abstract

**Background:**

Extended tunnelled roadways requiring ventilation via exhaust stacks are an increasingly common solution to traffic congestion around the world. In response to community concerns about adverse health effects associated with emissions from a new road tunnel exhaust stack, despite no demonstrable change in local ambient air quality, we conducted a cross sectional study to test for an association between exposure to the exhaust stack emissions and the presence of eye, nose and throat symptoms.

**Methods:**

Stack emissions were modelled and categorised into areas of high, medium and low levels of exposure to emissions. A telephone interview survey was conducted in these three zones. Multivariate analysis was undertaken using Cox Proportional Hazards modelling to estimate prevalence ratios between zones for eye, nose and throat symptoms.

**Results:**

The prevalence of eye, nose and throat symptoms in the study area were 50 percent, 67 percent and 33 percent respectively and did not differ between the exposure zones. The presence of these symptoms was associated with a measure of reported "environmental worry".

**Conclusion:**

The study did not demonstrate a community wide health impact associated with modelled emissions but is unable to exclude the possibility of sensitive individuals being adversely affected.

## Background

Extended tunnelled roadways requiring ventilation and exhaust stacks are a relatively new phenomenon in Australia. They have been built as one response to the need to alleviate traffic congestion within Sydney, Australia. Long tunnels require ventilation via exhaust stacks to maintain acceptable in tunnel air quality and avoid poorly dispersed emissions at tunnel portals. However, community concerns amongst residents in Sydney have arisen regarding exhaust stack emissions. There have been no previous studies directly addressing this concern.

Previous studies have examined health effects and environmental perception in communities around other point pollutant sources such as hazardous waste sites, waste incineration and industrial production [1-5]. The similarities in reported symptoms in these studies, regardless of the potential environmental hazard, are striking. All have found increases in eye, nose, throat or skin irritation or skin rashes in association with the point source. In particular, two studies reported significant associations between environmental worry and health symptoms [2,6]. This finding may go some way to explaining the contributing factors to health symptoms from environmental stressors. Traffic related air pollution has also been shown to be associated with respiratory symptoms such as wheezing, cough and allergic rhinitis [7,8] and sensitisation to pollen [9].

The M5 East motorway is a 10 km long, 4-lane dual carriage motorway, which links central Sydney with Sydney's southwest. Four kilometres of the M5 East motorway is a dual tunnelled section, which is ventilated via a single exhaust stack, located 900 metres north of the tunnelled motorway. The stack is situated in a valley with houses on a ridgeline overlooking the stack. The tunnels opened to traffic in December 2001 and are used by over 82 000 vehicles daily, with 6.9% being heavy vehicles [10].

In the first half of 2002, immediately after the opening of the M5 East tunnels, the New South Wales Health Department (NSW Health) received over 80 complaints from local residents who believed their health was being adversely affected by the M5 East stack exhaust. Continuous monitoring in the local area during the 12 months before the tunnel opened and up to 18 months after the tunnel opened showed no change in fine particle (measured as particulate matter less than 10 microns in diameter [PM_10_]), nitrogen dioxide or carbon monoxide concentrations. Selected air toxics (benzene, 1,3-butadiene, formaldehyde and acetaldehyde) were also collected every six days, with no apparent change in concentrations after the opening of the tunnels.

An exploratory, qualitative study [11] was undertaken in April and May 2003 to characterize the symptoms being reported and the nature of any apparent association with the M5 East stack. Residents living within 700 metres of the stack and those who made a complaint to NSW Health were invited to undergo a clinical assessment by a panel of physicians. The panel included physicians with expertise in allergy and immunology, respiratory medicine, occupational health and paediatrics. In addition, participants completed a health status questionnaire, had skin prick testing to common environmental allergens, and spirometry to assess lung function. On the basis of the overall evaluation, the panel classified subjects according to whether their symptoms were 'likely', 'uncertain' or 'unlikely' to be related to the M5 East stack. This qualitative investigation identified eye, nose, and throat irritation as the symptoms most likely to be associated with the M5 East stack. Respiratory symptoms, a common end point in air pollution literature, were not reported by the majority of participants in this qualitative study, and when reported were not related in time or place to potential stack emissions.

This paper describes a cross sectional study examining the association between exposure to stack emissions and the presence of the symptoms that were identified in the previous qualitative study.

## Methods

A cross-sectional study was undertaken within a 6 km × 6 km region centred on the M5 East stack. Level of exposure to emissions to the stack was assigned as high, medium or low based on location within this region. Symptoms were assessed by telephone interview survey.

### Assignment to exposure zones

Emission exposures zones were constructed within the study region using estimates of ground level concentrations of oxides of nitrogen (NOx) derived from stack and portal monitoring systems, and meteorological data collected at local air quality monitoring stations. NOx concentrations were used, as they are a good proxy for vehicle emissions [12]. These data were analysed using The Air Pollution Model (TAPM) version 2.3 [13].

TAPM is an air pollution modelling package, validated for this use, which integrated stack emission data with meteorological, soil type and terrain data to derive ground level concentrations. Grid spacing for predicted concentrations was 150 m.

The modelled ground level concentrations were used to delineate three exposure zones within the study area, using ERMapper v6.4 image processing and enhancement software [14]. NOx annual averages were used in the delineation of the sampling zones as the qualitative study identified that reported symptoms tended to be constant while at home, did not vary with season and were temporally related to the opening of the M5 East stack.

**Figure 1:**
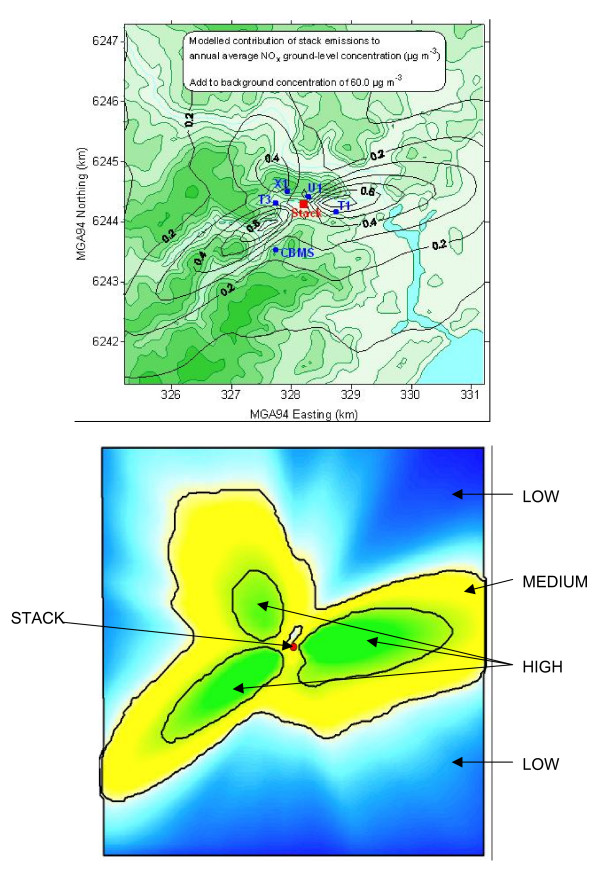
**Modelled annual concentrations of oxides of nitrogen from stack emissions, and February 2002 – January 2003 sampling zones**. (Upper figure). ---------(Black line) incremental annual NOx ug/m^3 ^above averaged background of 60 ug/m^3^. ---------(Green line) topographic contours. T1, U1, X1, T3, CBMS – Air monitoring stations. Red dot – Stack location.

**Figure 2:**
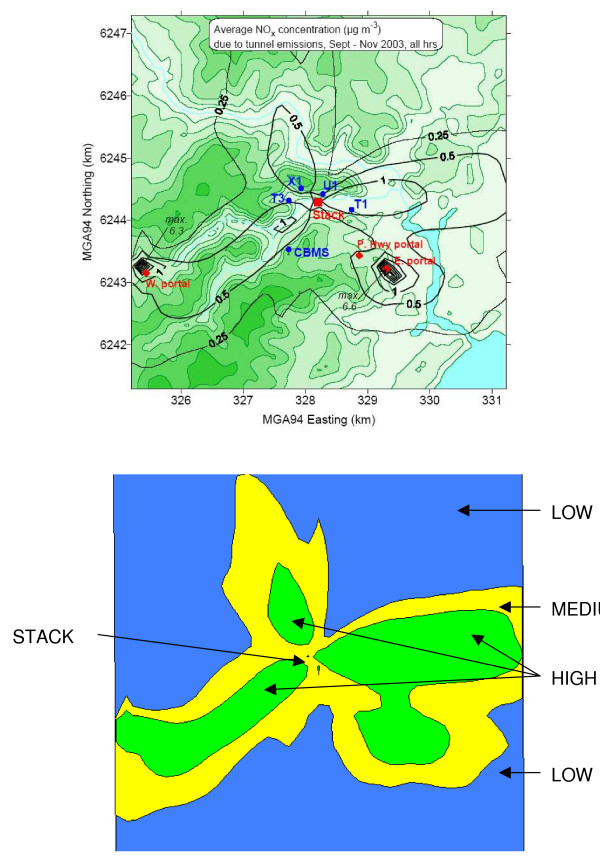
**Modelled average concentrations of oxides of nitrogen from stack and portal emissions (September – November 2003), and post hoc exposure zones**. (Upper figure). --------(Black line) incremental period average NOx ug/m^3 ^above averaged background. --------(Green line) topographic contours. T1, U1, X1, T3, CBMS – Air monitoring stations. Red dots – Stack and portal locations.

To select households for participation in the survey, all households in the study area were assigned to a census collector district and the collector district was assigned to a zone based on the location of the centroid of the collector district. Three zones were arbitrarily defined so that each zone contained enough residential households to ensure an adequate sample size to detect an effect. Relatively high, medium and low zones for recruitment were defined by annual mean NOx concentrations (February 2002–January 2003) of > 0.36 μg/m^3^, 0.36 – 0.20 μg/m^3 ^and < 0.20 μg/m^3^, respectively (Figure 1). However, the subsequent report of portal emissions occurring during the study period meant that the exposure zones for the analysis were re-assigned according to emissions during the study period, September to November 2003, using recorded emission data from the stack and the tunnel portals. The cutpoints for these post-hoc exposure zones were 0.54 μg/m^3 ^and 0.30 μg/m^3^, respectively (Figure 2).

### Study population and survey methods

The study population for the survey was all residents over the age of 17 years living in households with private telephones within the three initially identified exposure (sample) zones. Addresses from the electronic telephone pages were geocoded and assigned to each of the three sample zones using Mapinfo MapMarker v8.0 [15] and MapInfo Professional v6.5 software [16]. The resulting lists of residential telephone numbers for each sample zone were randomly sorted and supplied to the NSW Health Computer Assisted Telephone Interview (CATI) facility for interview.

Telephone interviews were undertaken using the NSW Health Survey methodology [17]. Household were randomly sampled and those selected were advised that they were selected to take part in a "local health survey". The number of persons occupying the household who were over 17 years old was determined then one person was randomly selected to take part in the study. If the person selected was unavailable, an appointment was made to speak with that person at a later date. Up to five calls were made in order to contact the selected respondent.

Telephone interviews were conducted by the NSW Health Survey CATI facility from October 1 to November 18 2003. As questions on the symptoms of interest related to the participants' experience in the previous four weeks, the telephone survey assessed symptomatology during Spring 2003 (that is, September to mid-November). The questionnaire was administered in the dominant languages of the area and a maximum of seven attempts per household were undertaken.

The questionnaire sought information on the principal outcome measures, potential confounders and effect modifiers of the association between the exposure and principal outcomes.

Principal outcome measures, defined on the basis of the qualitative study, were eye, nose and throat symptoms. Any reported symptom prompted further questions to assess frequency and severity. A symptom was defined as "frequent or severe" if, in response to further questions, the subject stated it was present often or constantly, or if he or she classed it as moderate or severe. Potential confounders were age, sex, exposure to cigarette smoke and other indoor pollutant sources such as attached garaging or unflued heating. Potential effect modifiers were age, general health, current asthma and the proportion of time spent at home. Questions on demographic and household characteristics, general health (Dartmouth Coop Function charts) and mental health (Kessler 6), chemical sensitivity, smoking status, environmental tobacco smoke exposure, garaging of vehicle, home heating and asthma were the same as those used in the NSW Health Survey [18]. Questions on eye symptoms were developed using the McMonnies Dry Eye questionnaire [19] and questions on environmental worry were adapted from Lipscomb et al. and Shusterman et al. [2,6] Environmental worry questions enquired into the participants' concern about environmental hazards in their neighbourhood. Participants were invited to rate their level of worry in terms of very, somewhat or not at all, and then specify if they felt these environmental hazards had affected their health. The research team developed questions on nose and throat symptoms. Teeth and gum symptom questions were modified from Lipscomb et al. [6] and designed to detect measurement bias from over or under reporting.

### Sample size

The estimated baseline prevalence of dry eyes was 10%. The sample size required (power = 80%, alpha = 0.05) to detect a difference of 6% or greater in the prevalence of dry eyes between two exposure zones was 524 in each exposure zone, that is 1572 in total [20].

### Analysis

Data were weighted to adjust for the selection probability of individuals within households. In addition, post-stratification weights were used to adjust for differences between the age-sex distribution of the respondents and that of the target population for each exposure zone [21].

Design-based analyses were conducted to account for features of the sample design and provide approximately unbiased estimates and appropriate standard errors [22,23]. Analyses were conducted using SAS v.8.0 and SUDAAN 8.0.1 statistical packages [24].

The following six outcomes were defined *a priori *to examine the association between exposure and symptoms:

1. Any eye symptom (soreness, scratchiness, dryness, grittiness, burning or watering) vs no eye symptoms

2. Any nasal symptom (itchiness, sneezing, dryness, runniness or congestion) vs no nasal symptoms

3. Any throat symptom (soreness or dryness) vs no throat symptoms

4. Frequent or severe eye symptoms vs others

5. Frequent or severe nasal symptoms vs others

6. Frequent or severe throat symptoms vs others

Associations between post-hoc exposure zones and the prevalence of symptoms were examined by estimating prevalence ratios, with 95% confidence intervals, using Cox's proportional hazards models with constant follow up time. This approach was used since the odds ratio, a measure of effect derived from logistic regression models, is a poor estimate of the risk ratio with common outcomes (> 10%) [25,26].

The following covariates were included in the multivariable analysis as clinically relevant, potential confounders: sex, age in years (continuous scale), exposure to cigarette smoke (yes/no), and potential exposure to motor vehicle emissions by having a garage internally connected to the home (yes/no). Current asthma (yes/no), general health (excellent, very good, good/fair, poor), time spent at home (most of the time/not most of the time) and age were tested for effect modification. No effect modification by the above factors was detected. A separate analysis was also conducted examining the association between environmental worry and the six symptoms using the same confounders as those described above.

Responses to the questions from the McMonnies Dry Eye Questionnaire were scored according to standard methodology [19]. Subjects with a score greater than 11 were classified as having dry eyes. We omitted two questions worth 4 points from standard questionnaire and so adjusted the published threshold down from 14.5 based on our judgement.

## Results

Telephone contact was made with 2433 eligible households within the study area and 1429 interviews were conducted with eligible participants (59% participation rate). The number of participants in each post hoc exposure zone was: high exposure zone 410 subjects, medium exposure zone 486 subjects, and low exposure zone 533 subjects. Seventy five interviews (5.2%) were conducted in a language other than English. The age and education level of participants did not differ between the zones (Table 1).

**Table 1:** Demographic characteristics by exposure zone

	High Zone (n = 410)	Medium Zone (n = 486)	Low Zone (n = 533)	Overall (n = 1429)
Age – Mean (years)	45	47	44	44
(95% CI)	(41 – 48)	(44 – 49)	(42 – 54)	(43 – 46)
Sex (Female%)	53.0%	46.6%	49.8%	49.3%
Home Ownership	63.5	66.7	58.7	61.1
(95% CI)	(55.2–71.8)	(59.7–73.7)	(53.3–64.1)	(57.0–65.1)
Highest Education Level Achieved				
Year 10 (%)	26.2	31.0	22.9	25.2
(95%CI)	(19.3–33.1)	(24.4–37.7)	(18.4–27.3)	(21.7–28.7)
Completed high school (%)	24.9	21.5	23.2	22.9
(95%CI)	(17.3–32.5)	(15.2–27.8)	(18.4–28.1)	(19.3–26.5)
Technical qualification (%)	21.3	21.7	23.1	22.7
(95%CI)	(15.6–26.9)	(16.1–27.2)	(18.7–27.6)	(19.4–25.9)
University (%)	27.7	25.8	30.8	29.2
(95%CI)	(20.3–35.1)	(20.3–31.4)	(25.7–35.9)	(25.5–33.0)

CI = Confidence interval

Subjects in the high exposure zone were more likely to be aware of its existence (Table 2). There were no differences in the prevalence of dry eyes between the three exposure zones (Table 3). The prevalence of 'any eye symptom' was higher in the medium exposure zone when compared to the low exposure zone but the prevalence of this symptom was not significantly higher in the high exposure zone compared to the low exposure zone (Table 3). There were no significant differences in the prevalence of any other symptoms between the exposure zones (Table 3).

**Table 2:** Selected respondent characteristics by exposure zone

	High Exposure Zone % (95%CI)	Medium Exposure Zone % (95%CI)	Low Exposure Zone % (95%CI)	Total % (95%CI)	New South Wales % (95%CI)
General Health^a^	84.7	76.7	81.5	80.5	80.7
	(80.5 – 89.0)	(70.7 – 82.7)	(77.8 – 85.2)	(77.6 – 83.4)	(79.7–81.7)
Psychological Distress^b^	9.2	15.2	13.2	13.3	12.2
	(5.8–12.7)	(9.6–20.7)	(9.7–16.7)	(10.6–16.1)	(11.4–13.1)
Current Asthma^c^	6.8	9.9	11.6	10.8	10.6
	(4.0–9.7)	(4.8–15.0)	(8.1–15.1)	(8.1–13.4)	(9.8–11.3)
Environmental Worry (very worried)	15.8	13.7	11.8	12.6	N/A
	(11.1–20.4)	(9.8–17.7)	(8.4–15.2)	(10.1–15.1)	
Exposure Time (most time spent at address)	49.1	51.5	49.6	50.1	N/A
	(41.2–57.0)	(44.5–58.5)	(44.3–54.9)	(46.1–54.1)	
Foreign odour detected in past few days or past week	29.2	24.2	23.4	24.1	N/A
	(21.6–36.9)	(17.7–30.7)	(19.2–27.7)	(20.8–27.4)	
Awareness of survey link to M5 stack	12.6	10.8	2.2	5.2	N/A
	(8.8–16.5)	(7.4–14.2)	(1.0–3.3)	(4.0–6.4)	
Unflued gas or solid fuel heater	27.1	25.9	28.4	27.6	22.6
	(20.3–33.9)	(19.7–32.0)	(23.4–33.4)	(23.9–31.3)	(20.6–24.7)
Personal Smoking (daily or occasionally)	17.9	24.8	26.6	25.4	21.4
	(12.2–23.7)	(18.5–31.1)	(21.9–31.3)	(21.9–28.9)	(20.3–22.4)
Smoke Free Households	82.0	85.5	78.9	80.8	81.0
	(76.3–87.8)	(80.5–90.4)	(74.5–83.3)	(77.6–84.0)	(80.0–82.0)
Garage with direct access to home	11.3	8.4	11.8	10.9	22.2
	(6.1–16.5)	(5.0–11.8)	(8.2–15.3)	(8.4–13.5)	(20.0–24.3)
Diagnosed Chemical Sensitivity	1.4	3.6	2.4	2.6	2.9
	(0.3–2.6)	(0.4–6.8)	(0.7–4.0)	(1.2–4.0)	(2.5–3.4)
Any Teeth and/or Gums soreness	18.5	20.1	19.0	19.2	N/A
	(12.5–24.5)	(14.8–25.4)	(14.6–23.5)	(16.0–22.5)	

CI = Confidence interval

^a^General health rated as excellent, very good or good

^b^Kessler 6 scored at high or very high psychological distress.

^c^Been told by a doctor or at a hospital that they had asthma AND had symptoms of asthma or taken treatment for asthma in the past 12 months.

**Table 3:** Symptom prevalence and adjusted prevalence ratio by exposure zone

Symptom	Exposure Zone	Prevalence % (95% CI)	Adjusted prevalence ratio^a ^(95% CI)
Dry Eye	High	7.1 (3.8 – 10.5)	1.12 (0.62 – 2.02)
	Medium	7.5 (3.4 – 11.5)	1.11 (0.59 – 2.06)
	Low	6.3 (3.9 – 8.7)	1.00
	Overall	6.6 (4.7 – 8.6)	

Any Eye Symptom	High	51.6 (43.8 – 59.5)	1.11 (0.92–1.34)
	Medium	57.3 (50.6 – 64.1)	1.23 (1.05–1.45)
	Low	47.1 (41.8 – 52.4)	1.00
	Overall	50.0 (46.0 – 54.0)	

More Frequent and/or Severe Eye Symptom	High	15.0 (10.2 – 19.7)	0.95 (0.64–1.40)
	Medium	21.3 (15.4 – 27.1)	1.28 (0.89–1.83)
	Low	16.0 (12.2 – 19.8)	1.00
	Overall	17.2 (14.2 – 20.1)	

Any Nasal Symptom	High	60.3 (52.4 – 68.2)	0.90 (0.77–1.04)
	Medium	67.4 (61.0 – 73.9)	1.01 (0.89–1.14)
	Low	67.2 (62.2 – 72.2)	1.00
	Overall	66.6 (62.9–70.4)	

More Frequent and/or Severe Nasal Symptom	High	30.8 (24.0 – 37.7)	1.00 (0.76–1.31)
	Medium	38.2 (31.4 – 45.0)	1.20 (0.96–1.52)
	Low	31.2 (26.5 – 36.0)	1.00
	Overall	32.9 (29.2–36.6)	

Any Throat Symptom	High	31.0 (23.4 – 38.6)	0.96 (0.73–1.28)
	Medium	32.9 (26.5 – 39.3)	1.01 (0.79–1.30)
	Low	33.5 (28.4 – 38.6)	1.00
	Overall	33.1 (29.3–36.9)	

More Frequent and/or Severe Throat Symptom	High	13.7 (9.0 – 18.4)	0.97 (0.63–1.49)
	Medium	15.7 (10.7 – 20.6)	1.07 (0.72–1.59)
	Low	14.8 (11.1 – 18.5)	1.00
	Overall	14.9 (12.1–17.7)	

CI = Confidence interval

^a^Adjusted for age, sex, cigarette smoke and internal garaging.

There was a significant association between subjects' worry about environmental effects on their health and the presence of all six symptom outcomes (Table 4). There was no association between exposure zone and worry.

**Table 4:** Adjusted prevalence ratio of environmental worry by symptom

Symptoms	Environmental Worry^a^	
	Somewhat (95% CI)	Very (95% CI)

Any Eye Symptom	1.10 (0.91 – 1.32)	1.55 (1.30 – 1.86)
Frequent and/or Severe Eye Symptom	1.10 (0.75 – 1.61)	1.75 (1.14 – 2.69)
Any Nasal Symptom	1.24 (1.10 – 1.41)	1.24 (1.05 – 1.46)
Frequent and/or Severe Nasal Symptom	1.38 (1.07 – 1.77)	1.90 (1.43 – 2.54)
Any Throat Symptom	1.15 (0.89 – 1.48)	1.38 (1.02 – 1.88)
Frequent and/or Severe Throat Symptom	1.49 (0.98 – 2.26)	2.26 (1.36 – 3.75)

CI = Confidence interval

^a ^Reference = "not at all". Adjusted for age, sex, zone, cigarette smoke and internal garaging.

## Discussion

This study did not demonstrate consistent associations between modelled exposures to NOx emissions from the M5 East stack and self-reported eye, nose and throat symptoms. Environmental worry was significantly associated with all six symptom outcomes.

The finding of a higher prevalence of 'any eye symptom' in the medium exposure zone compared with the low exposure zone is difficult to interpret. There was no difference in prevalence between low and high exposure zones. The absence of a dose-response effect means that a clinically important adverse effect is unlikely. The apparent association may be a false positive finding (Type I error) made more likely by multiple comparisons (14 comparisons between zone and symptoms were undertaken).

The study did not include a control group from an area entirely remote from the M5 East stack. Instead, we relied on using the lower exposure zone as a reference area and compared symptoms from the medium and high zones to this area. This was done to limit variability in other potentially confounding factors, such as background ambient air pollutant levels and geographic specific factors (e.g. ethnicity, socio-economic status).

Children were not included in this survey as there were too few children who presented for assessment in the qualitative phase to formulate a case definition for children. Hence, we cannot draw conclusions about potential adverse effects of stack emissions on children.

The study was designed to be able to detect a 6% or greater difference in the prevalence of dry eyes between two of the exposure zones with a power of 80%. The actual power of the study to detect this difference was approximately 60%. The study had 80% power to detect a difference of 8% as significant. This modest decrease in power, due to the re-allocation of some subjects from the original exposure sampling zones to post-hoc exposure zones, is justifiable as the post-hoc zones were a better representation of exposure during the study period.

Exposure assessment for this survey was based on modelled data for ground level exposure within the study area. A range of alternative methods of exposure assessment were considered. Personal monitoring was not feasible since it would have been necessary to monitor a range of pollutants over an extended time period to get representative data, relevant to the symptom questionnaire. Distance from the point source of exposure, that is the exhaust stack, was also a potential alternative exposure measure [27]. However, we considered this would not be adequate due to the complex nature of the topography in the study area. Average pollutant concentrations, rather than peak concentrations, were used to allocate exposure zones. This was because the qualitative study identified health effects that did not vary seasonally or day to day.

The estimated increase in levels of pollutants above background levels is approximately one percent. Such a small gradient of NOx concentration is unlikely to be responsible for an increase in symptoms. It has been postulated that if health effects were to be found, then other compounds that are not currently being monitored may cause them. However it is reasonable to assume that if these unknown compounds were traffic derived then they would be distributed in the same pattern as NOx, and hence NOx levels can be used as proxy measures for these unknown compounds.

Historical emission data were used as a basis for the stratified sampling technique with post-hoc emission data used to define exposure zone for the analysis of data. The exposure zones were very similar (figure 1 &2) and consequently it is unlikely that a sampling bias has been introduced by this approach.

Symptoms were assessed by means of a telephone survey methodology that is widely used and has been validated for the collection of health information at a community level [17]. Many of the questions used in this survey were taken from the NSW Health Survey enabling us to compare the survey population with the NSW population as a whole. To limit recall bias, respondents were asked about symptoms occurring in the preceding four weeks.

It is possible that subjects who were aware of the purpose of the study might have systematically over or under reported symptoms. While there was a significant difference in those who were aware of the study between zones, there was no significant difference in the self-report of "teeth and gums symptoms" (a variable used to determine over or under reporting) between zones. We therefore believe that such measurement bias is minimal. Selection bias may have occurred if those with unlisted telephone numbers, those without a telephone in their household or those who declined to participate in the survey were different from those who participated in the survey. Since we only interviewed current residents it is possible that our findings are biased by a "healthy-resident" effect. If residents experiencing symptoms have moved away from the area, this would bias the study population and may influence the findings of the cross-sectional survey.

Environmental worry was not included in our initial Cox regression models due to the concern that an individual with symptoms may be more likely to report being worried simply as result of being unwell. In a separate analysis, we included environmental worry as an independent variable. Our findings of an association between self reported symptoms and environmental worry confirm results from other similar studies [2,6]. In these studies, environmental worry was associated with one or more irritative symptoms (eye, nose, throat or skin irritation). While not specifically examining the relationship between environmental worry and environmental stress, other studies looking at the health effects of environmental stressors have also found significant eye, nose, throat or skin irritative symptoms [1,3-5].

## Conclusion

We found no population level association between the prevalence of reported symptoms and modelled emissions from the M5 East stack. This study cannot rule out the possibility that certain sensitive individuals do experience symptoms related to the stack. The implication of the observed association between symptoms and self reported environmental worry is uncertain. There is a growing body of evidence to suggest that communities surrounding an environmental stressor do experience greater self reported environmental worry and symptom reporting. Cohort studies, initiated before the implementation of large infrastructure projects, may be better placed to assess the health impacts of these projects than a cross sectional study as reported here. Improved risk communication may be an effective means to reduce environmental worry.

## Competing interests

The authors declare that they have no competing interests.

## Authors' contributions

AC contributed to study design and management, basic analysis and drafted the manuscript. VS contributed to study design and management. KI undertook statistical analysis. BJ contributed to study design and data analysis. MS contributed to study design and management. GM contributed to study design. AW undertook exposure analysis assignment. All authors helped draft, read and approved the final manuscript.
